# Proteomics Reveals Differential Diagnosis Biomarkers Between Sepsis and Hemophagocytic Syndrome

**DOI:** 10.3390/biomedicines13123113

**Published:** 2025-12-17

**Authors:** David Martin-Pestana, Mikel Azkargorta, Francisco Javier Pilar-Orive, Silvia Redondo, Janire Urrutia, Cristina Calvo, Felix Elortza, Itziar Astigarraga, Susana Garcia-Obregon

**Affiliations:** 1 Pediatric Oncology Group, Biobizkaia Health Research Institute, 48903 Barakaldo, Spain; dmape@birc.au.dk (D.M.-P.); janire.urrutia@ehu.eus (J.U.); itziar.astigarraga@osakidetza.eus (I.A.); 2 Proteomics Platform, CIC bioGUNE, Basque Research and Technology Alliance (BRTA), CIBERehd, Bizkaia Science and Technology Park, 48160 Derio, Spain; mazkargorta@cicbiogune.es (M.A.); felortza@cicbiogune.es (F.E.); 3 Pediatric Critical Care Service, Hospital Universitario Cruces, 48903 Barakaldo, Spain; jporive@outlook.com (F.J.P.-O.); silvia.redondoblazquez@osakidetza.eus (S.R.); 4 Pediatric Department, Hospital Universitario Cruces, 48903 Barakaldo, Spain; 5 Physiology Department, Faculty of Medicine and Nursing, Euskal Herriko Unibertsitatea UPV/EHU, 48940 Leioa, Spain; 6 Pediatric Critical Care Service, Hospital Universitario Donostia, 20014 San Sebastián, Spain; cristina.calvomonge@osakidetza.eus; 7 Pediatric Department, Faculty of Medicine and Nursing, Euskal Herriko Unibertsitatea UPV/EHU, 48940 Leioa, Spain

**Keywords:** hemophagocytic lymphohistiocytosis, sepsis, serum proteomics

## Abstract

**Background/Objectives**: Hemophagocytic Lymphohistiocytosis (HLH) shares many clinical features with sepsis. To improve HLH diagnosis and its differential diagnosis with sepsis, serum proteomic analyses of healthy donors, HLH and septic patients were performed. **Methods**: Twenty-four individuals were enrolled in a label-free MS/MS spectrometry analysis. STRING was conducted to study the protein–protein interactions overrepresented within the proteins of each comparison. To integrate the functions of the proteins with their respective regulation patterns, Ingenuity Pathway Analysis software was used. Validation of selected proteins was carried out by ELISA. **Results**: Proteomic results revealed 537 differentially expressed proteins (DEPs) between HLH and sepsis, 471 DEPs between HLH and healthy donors, and 37 DEPs between sepsis and healthy donors. These results were subjected to functional analysis, which showed that apart from inflammation and lipid metabolism, the proteostasis network was deeply impaired in the HLH condition. Considering this information, protein fold changes and the functions of six proteins were validated by ELISA. **Conclusions**: sCD300a, sCD300b and sCD25 could be specific serum biomarkers for HLH diagnosis, and SAA-1 and LRG1 might be useful biomarkers for differential diagnosis between sepsis and HLH. PSMB1, a non-catalytic subunit of the 20S proteasome, showed promising results for HLH-specific and differential diagnosis. Its elevation in HLH patients may reflect an intensified demand for protein turnover, possibly driven by a higher activation of the immunoproteasome. These insights contribute to expanding our understanding of HLH pathophysiology regarding new pathways and highlight innovative therapeutic interventions, such as Bortezomib and other next-generation inhibitors, designed to modulate immunoproteasome activity.

## 1. Introduction

Hemophagocytic lymphohistiocytosis (HLH) or hemophagocytic syndrome is a rare, life-threatening syndrome caused by an abnormally high activation of the immune system [1,2]. HLH occurs in both adult and pediatric patients, and the mortality is high at any age [2,3]. Due to the severity of the syndrome, it is crucial to recognize HLH and start its treatment as soon as possible. Specific HLH therapy is not well established currently, but identification and treatment of the trigger is needed, as well as systemic cytotoxic, immunosuppressive and other drugs to suppress the hyperinflammatory condition of the patient. Also, in genetic cases, allogeneic hematopoietic cell transplantation is often carried out [4,5]. Despite prompt recognition of HLH being so relevant, it is usually underdiagnosed as it shares many clinical features with other severe hyperinflammatory syndromes, mainly sepsis and septic shock [6]. However, due to the lack of specific biomarkers for HLH, the diagnosis is very challenging [5,6,7,8,9].

Sepsis is defined as a life-threatening organ dysfunction caused by a dysregulated host response to infection [10]. The current incidence is about 216 cases per million children per year in middle-to-high income countries [11]. Mortality is lower in pediatric sepsis than in HLH, ranging from 9% to 20% depending on both geographical location and presence of comorbidities [11]. Prompt recognition and treatment of sepsis with antimicrobial therapy is crucial for a better outcome of the patients [12]. However, sepsis does not have a standard validated diagnostic biomarker either, and its clinical resemblance to HLH has led to confusion between the two diseases [13]. Considering the differences in their treatments and the overlapping of clinical features, the prognosis of HLH patients is often worsened due to the delay in initiating correct therapy [2,6]. Therefore, identifying specific biomarkers for sepsis, HLH and differential diagnosis of both syndromes has become a priority in the clinical management of these groups of patients [2]. Recently, based on bibliography research, a consensus-based guideline for the prompt recognition of HLH in Intensive Care Units has been published. However, new approaches for the discovery of new differential diagnosis biomarkers are needed [14].

Serum protein profiling is a new valuable resource for biomarker discovery and understanding the pathogenesis of diseases [15,16]. For this reason, in this study we analyzed the serum proteome from HLH, septic and healthy individuals in order to ease the identification of new biomarkers for diagnosis of HLH and differential diagnosis between HLH and sepsis.

## 2. Materials and Methods

### 2.1. Sample Collection and Ethical Commitment

An observational case–control study was carried out at a tertiary hospital (Hospital Universitario Cruces, Bizkaia, Spain). For this study, peripheral blood samples from venous catheters of patients diagnosed with HLH or sepsis/septic shock and from pediatric healthy donors were collected at diagnosis and before starting treatment. HLH diagnosis was made according to HLH-2004 guidelines [7], and for sepsis/septic shock patients the Consensus Conference on Pediatric Sepsis Criteria were followed [10]. Patients who had recently received blood products and those with immunosuppressed conditions or with other immunological/inflammatory diseases were excluded. Controls were healthy children who required blood tests as part of preoperative study for minor surgery (groin hernia, etc.) or those without proven disease (constitutional growth delay, etc.). Patients were excluded from the control group if they had a fever or an acute inflammatory or inflammation disease. Demographics and clinical data for the cohorts of patients and healthy donors (age, sex, comorbidities and organ dysfunction) were collected.

Serum was extracted by centrifugation of blood samples at 2000 rpm for 20 min and stored at −80 °C at Basque Biobank for Research-OEHUN until analysis. Extraction and sample processing were performed based on a standardized protocol.

This project was approved by the Research Ethics Committee at Hospital Universitario Cruces (CEIC E10/50) and the Basque Ethics Committee for Clinical Research (PI2012165 and PI 2020036). All the patients and/or parents/legal guardians signed informed consent forms. The study was performed according to Spanish Law (Biomedical Research and Protection of Personal Data) and the Declaration of Helsinki.

### 2.2. Study Endpoints

The primary endpoint of this study was the difference in serum protein abundance between patients suffering from HLH and those with sepsis/septic shock, with the aim of identifying biomarkers that could aid in the differential diagnosis between these two conditions. Secondary end-points included (i) the functional characterization of proteins differentially expressed between HLH and sepsis/septic shock patients and healthy donors; (ii) the validation of selected candidate proteins by ELISA in a larger cohort of patients and healthy donors; and (iii) the evaluation of the diagnostic performance of these proteins for HLH and sepsis diseases using receiver operating characteristic (ROC) curve analysis (area under the curve, sensitivity and specificity).

### 2.3. Proteomic Study

For the proteomic study, serum samples from eight HLH, eight sepsis/septic shock and eight healthy donors were selected, for a total of twenty-four samples. Pierce Top 12 Abundant Protein Depletion Spin Columns (Thermo Scientific, Walthman, MA, USA) were used with 10 μL of serum samples to deplete those abundant proteins and enhance the detection of less-abundant proteins. Samples were digested following a filter-aided sample preparation (FASP) protocol [17]. Samples of 200 ng were loaded onto an Evosep One chromatograph (Evosep, Odense, Denmark) and eluted peptides were analyzed in a novel hybrid trapped ion mobility spectrometer (timsTOF Pro with PASEF, Bruker Daltonics, Billerica, MA, USA). Database searching was performed using PEAKS (Bioinformatics Solutions Inc., Waterloo, ON, Canada) against a Homo sapiens Uniprot/Swissprot database with precursor and fragment tolerances of 20 ppm and 0.05 Da. Abundances of proteins identified with at least two peptides at false discovery rate (FDR) < 1% were loaded onto Perseus software version 1.6.14.0 for statistical analysis. Transformation by logarithm of base 2 and selection of proteins present in ≥70% of the samples of at least one of the groups were performed. Missing values were imputed with 10% of the lowest values of the distribution for each sample and a quantile normalization was applied. The resulting abundances were tested for significance between groups with an ANOVA with Tukey HSD test. Proteins with *p* value < 0.05 in both tests were selected. The mass spectrometry proteomics data have been deposited into the ProteomeXchange Consortium via the PRIDE [18] partner repository with the dataset identifier PXD063444.

**Figure 1:**
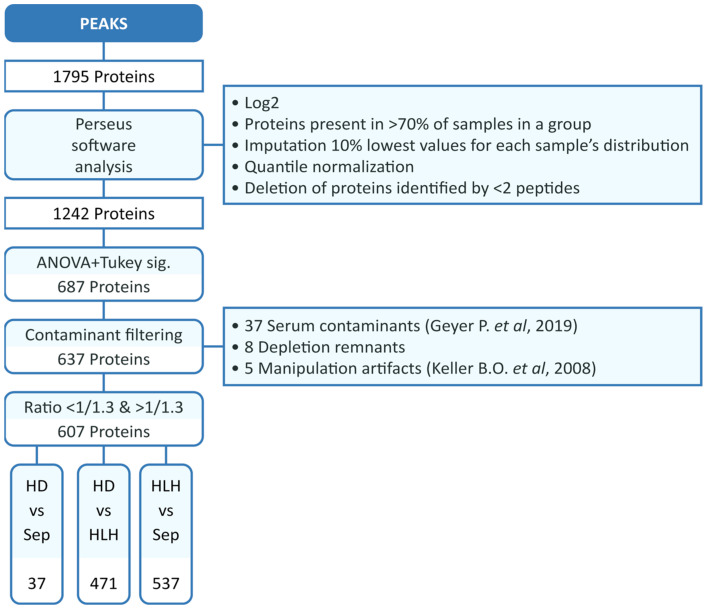
HLH patients present significantly more DEPs in serum than septic patients. Diagram of DEPs in the proteomic study. DEPs: differentially expressed proteins. HD: healthy donors; Sep: septic patients; HLH: hemophagocytic lymphohistiocytosis [19,20].

Serum contaminants [19], manipulation artifacts [20] and depletion remnants were excluded from the subsequent analysis. Finally, a cutoff was applied with the aim of tracking biologically relevant subtle changes, so only those proteins with fold change (FC) above 1.3 in either direction were considered for further analyses (see Figure 1).

### 2.4. Analysis of the Serum Proteome Profile

To understand the expression pattern of differentially expressed proteins (DEPs), volcano plots were created using the tool SRplot (https://www.bioinformatics.com.cn, accessed on 17 June 2023) with previously described significant cutoff values. The same tool was used to create both Venn diagrams (to analyze the overlapping among the different comparisons) and a heatmap (for checking whether the serum proteome signature can be clustered into septic and HLH groups). For plotting the heatmap, the ratio used was obtained by comparing the abundance of each protein for each patient and the average abundance of the same protein in healthy donors.

Next, a search using the STRING database (http://www.string-db.org/, accessed on 17 June 2023) was conducted to study the protein–protein interactions (PPIs) overrepresented within the proteins of each comparison. The analysis was performed with the highest confidence setting (0.9), and with neither text mining nor co-occurrence, as the first one is prone to introduce false positives, while the second one is used when more than one species is analyzed. Finally, an unsupervised Markov Cluster Algorithm (MCL) clustering method with an inflation parameter of four was computed for each STRING representation to group the PPIs according to their main Gene Ontology (GO) terms.

To integrate the functions of the proteins with their respective regulation patterns, Ingenuity Pathway Analysis (IPA) software (version IPA Fall Release Oct 2025; Qiagen, Hilden, Germany) was used. Proteins were uploaded to the software with thresholds of 0.05 for the pairwise-adjusted *p* value (Tukey test) and 1.3 of FC in either direction. A graphical summary and impaired pathways were analyzed for each of the three comparisons (septic versus healthy donors, HLH versus healthy donors, and HLH versus septic samples).

### 2.5. Selection of Proteins for ELISA Validation

To validate useful biomarkers for diagnosis of HLH and differential diagnosis between HLH and sepsis in a larger cohort of patients and healthy donors, some steps were followed: First, a bibliographic search was conducted. Function, disease association and condition as biomarkers in either sepsis or HLH for the deregulated proteins were documented. Only proteins significant in comparisons including HLH (HLH versus healthy donor and HLH versus sepsis) were analyzed. Considering this information, FC values, imputation data and commercial ELISA kit availability, the following proteins were selected for validation: sCD25, sCD300a (R&D Systems, Minneapolis, MN, USA), sCD300b (Wuhan Fine Biotech Co., Wuhan, China), PSMB1 (MyBioSource, San Diego, CA, USA), LRG1 (Invitrogen, Waltham, MA, USA) and SAA-1 (Cloud-Clone Corp. CCC, Katy, TX, USA). ELISA assays were carried out following the protocols of each manufacturer. Due to sample size and the non-normal distribution of the data, comparisons among groups were performed using a two-step non-parametric approach: first, a Kruskal–Wallis test was applied to evaluate overall differences among the three groups (HLH patients, septic patients and healthy donors); second, when the Kruskal–Wallis test was significant, post hoc pairwise comparisons were performed using Mann–Whitney U tests with Bonferroni correction. An adjusted *p*-value < 0.05 was considered statistically significant.

In addition, to study the discriminatory potential of the validated proteins for HLH disease and sepsis, we created receiver operating characteristic (ROC) curves using “pROC” R statistical package [21].

## 3. Results

### 3.1. Characteristics of the Cohort

Eight HLH patients (five males and three females; median age: 2.39 years old), eight septic patients (five males and three females; median age: 3.8 years old) and eight healthy donors (six males and two females, median age: 6.1 years old) were included in the proteomic study. Familial HLH was detected in five children, whereas in three no mutation was found; all of them were triggered by a virus infection (EBV, CMV, enterovirus or VIH). Among HLH patients, the mortality was very high: there was a unique survivor; seven of them died. One sepsis patient and seven septic shock patients were diagnosed in the septic group, but infections were confirmed by microbiological studies in only two children. Seven of them developed multiorgan failure (three suffered dysfunction of two or more organs). Nevertheless, seven of them survived and one died. A larger cohort of similar sex and age characteristics was used for validation, consisting of 21 healthy donors, 37 sepsis/septic shock patients and 15 HLH patients. The features of the individuals included in the study are shown in Table 1.

### 3.2. Expression Analysis of the Serum Proteome Profile

**Figure 2:**
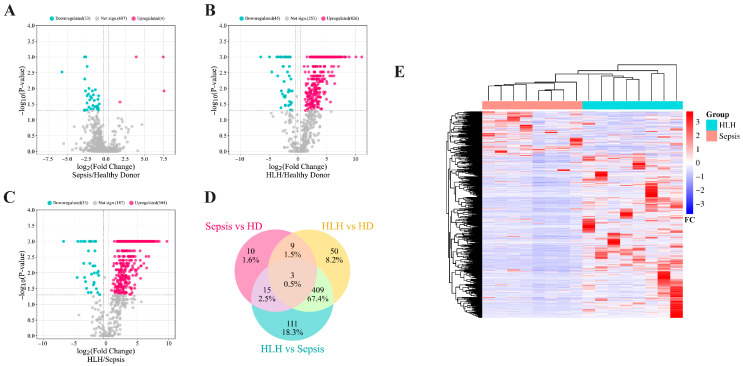
The proteome of the HLH group greatly differs from that of healthy donors (HD) and the septic group. Volcano plots of differentially expressed proteins between (**A**) septic patients and healthy donors; (**B**) HLH patients and healthy donors; and (**C**) HLH patients and septic patients. (**D**) Venn diagram of deregulated proteins in sepsis versus healthy donors, HLH versus healthy donors and HLH versus sepsis. (**E**) Heatmap representation of 1242 proteins recognized in the proteomic study. Each column shows the serum proteome expression profile of each patient relative to healthy donors. Septic patients are in the light red group, while HLH patients appear in the light blue group.

A significantly different serum proteome signature was detected between septic patients, HLH patients and healthy donors. As shown in Figure 1 and Figure 2A, 37 proteins appeared to be deregulated when comparing septic patients and healthy donors. HLH patients showed a much deeper deregulation, with 471 total proteins deregulated when compared to healthy controls (Figure 2B), and 537 proteins were deregulated when compared to septic patients (Figure 2C). Both of the latter comparisons showed a prominent upregulation pattern in favor of HLH. Moreover, in Figure 2D the overlapping of proteins in different diseases is represented; it was remarkable that an average of 70% of results overlapped when comparing the significantly deregulated serum proteins between HLH versus healthy donors and HLH versus the septic group.

The heatmap (Figure 2E) showed a clear differentiation between HLH and septic groups, as there was a notable number of proteins upregulated in HLH patients that were not deregulated or slightly downregulated in the septic condition.

### 3.3. Functional Analysis of the Serum Proteome Profile

**Figure 3:**
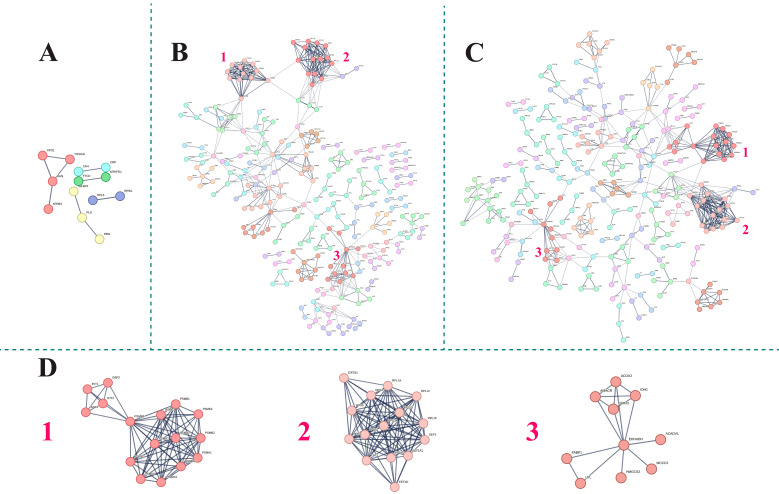
Main overrepresented clusters in HLH versus sepsis and healthy controls are formed of proteasome components (D1), ribosomal proteins (D2) and lipid metabolism enzymes (D3). STRING representation of differentially expressed proteins in (**A**) septic group versus healthy donors; (**B**) HLH patients versus healthy donors; and (**C**) HLH versus septic patients. (**D**) Most overrepresented interaction networks in both HLH versus healthy donors and HLH versus septic patients.

Functional interactions of deregulated proteins in each pathological condition are shown in Figure 3. Figure 3A represents interactions of proteins deregulated in septic patients’ serum when compared to healthy donors. The results exhibited in Figure 3B,C show that the relationships among deregulated proteins when comparing HLH to both healthy controls and septic patients, respectively, were more complex. The three most overrepresented clusters in both comparisons involving the HLH condition were the same and are shown in Figure 3D.

The analysis identified several deregulated processes related to protein turnover and synthesis, as well as lipid metabolism (see Table 2). Proteolytic pathways (Figure 3D(1)), including the proteasomal ubiquitin-independent protein catabolic process (GO:0010499) and regulation of cellular amino acid metabolic processes (GO:0006521), were notably affected. Concurrently, key functions involved in protein synthesis also exhibited altered regulation (Figure 3D(2)), mainly SRP-dependent cotranslational protein targeting to the membrane (GO:0006614) and translational initiation (GO:0006413). In addition to these protein-related pathways, fatty acid β-oxidation using acyl-CoA oxidase (GO:0033540) and protein targeting to peroxisomes (GO:0006625) were found to be disrupted, indicating changes in fatty acid metabolism (Figure 3D(3)).

To study the dysregulated functions of HLH and septic patients through the serum proteome data (Figure 2), Ingenuity Pathway Analysis (IPA) software was used. The number of differentially expressed proteins between septic and healthy donor groups was not enough for IPA software to either create a summary graphic or to make any z-score prediction on the regulation pattern of the associated pathways. However, the larger number of proteins in the HLH comparison against healthy donors and septic patients allowed IPA to output more solid results and predictions. For this reason, the analysis was focused on the HLH versus healthy donors (to learn about defective mechanisms in HLH) and HLH versus septic patients (to identify differential characteristics of both syndromes).

**Figure 4:**
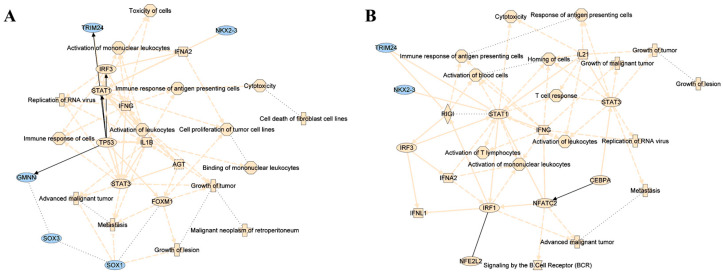
HLH shows a distinguishable inflammation profile compared to both healthy and septic individuals. Graphical summary of pathways and functions predicted to be upregulated (yellow) or downregulated (blue) when comparing (**A**) HLH serum proteome to healthy donors and (**B**) to septic patients.

Among the deregulated pathways and functions identified in both comparisons, it was remarkable to observe the extensive proinflammatory signature of HLH. The main upregulated pathways when comparing HLH to healthy individuals (Figure 4A) comprised different types of IFN, (IFNγ and IFNα2), IRF3, STAT1/3, and some types of interleukin signaling (IL-1β). Some translation and transcription regulators were also upregulated, like TP53 and FOXM1, while others appeared downregulated, like TRIM24, NKX2-3, GMNN, SOX3 and SOX1. In addition, some pathways related to the immune system, such as activation of mononuclear leukocytes, immune response to antigen-presenting cells, cytotoxicity, binding of mononuclear leukocytes, and immune response of cells, were upregulated.

When analyzing the differential characteristics between the HLH serum proteome signature and that of septic patients (Figure 4B), the general proinflammatory signature was still quite notable. Although some of the previously described pathways and regulators were still present with the same pattern, such as IFN-γ, IFNα2, STAT1/3, IRF3, TRIM24 and NKX2-3, others like IL-21 IRF1, IFNλ1, CEBPA and NFATC2 also appeared upregulated in this case. Moreover, some of the processes related to the immune system, such as cytotoxicity and the activation of leucocytes and lymphocytes, were also upregulated when the HLH serum proteome was compared with septic patients.

**Figure 5:**
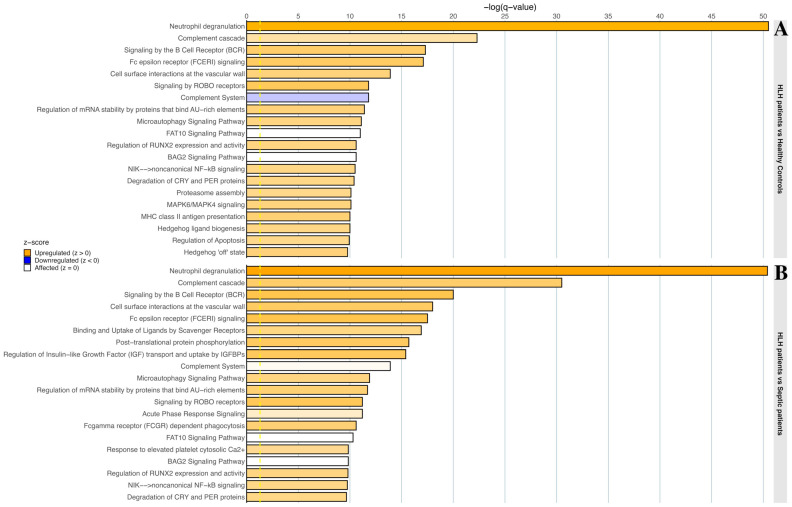
Pathways predicted to be deregulated between (**A**) HLH patients and healthy donors and (**B**) between HLH patients and septic patients. Bar color intensity correlates with z-score magnitude.

When studying the top 20 most significantly (*q*-value < 0.05) dysregulated pathways in the HLH disease versus healthy donor comparison (Figure 5A), 14 overlapped with those identified in the HLH disease versus sepsis comparison (Figure 5B), reflecting similarities in the deregulated serum proteome between both contrasts. To compare the magnitude and direction of regulation between these two comparisons, the pathway activation *z*-score was used as an effect-size measure (with positive values indicating upregulation and negative values indicating downregulation). In the HLH disease versus sepsis comparison (Figure 5B), a positive *z*-score therefore indicates that a given pathway is more activated in HLH disease than in septic patients.

The overlapping pathways could be grouped into two main clusters. Firstly, a group is directly related to the immune system. Within this group, “binding and uptake of ligands by scavenger receptors” and “acute phase response signaling” showed positive activation *z*-scores in the HLH versus sepsis comparison, indicating stronger upregulation in patients diagnosed with HLH than in septic patients. Secondly, the proteostasis network was notably altered, with BAG2 signaling and FAT10 signaling appearing over-represented in the analysis. Moreover, lipid metabolism was impaired in HLH disease when compared with healthy donors and between HLH disease and sepsis. Supplementary Table S1 extends the analysis beyond the top 20 pathways displayed in Figure 5.

### 3.4. Validation of Candidate Biomarkers

For ELISA validation, six proteins were selected (SAA-1, LRG1, PSMB1, sCD300a, sCD300b, and sCD25) in a larger cohort (21 healthy donors, 37 sepsis/septic shock patients and 15 HLH patients). Among these, the first three proteins (SAA-1, LRG1 and PSMB1) were chosen based on the proteomic analysis. SAA-1, a classical acute-phase reactant, was selected because in our proteomic data it showed upregulation in sepsis compared with both healthy donors and HLH, while remaining largely unchanged between HLH and healthy donors, suggesting its value as a differential marker for both diseases. LRG1, which participates in innate immune and inflammatory pathways, was also differentially expressed in the study cohort and had a robust, commercially available serum ELISA, making it suitable for validation. Lastly, PSMB1 was prioritized as a representative proteasome subunit because our functional analyses indicated proteasome involvement in HLH and because the proteasome is a target of existing immunomodulatory therapies such as bortezomib [22].

In addition, sCD300a and sCD300b were included to explore the potential involvement of the CD300 family in HLH and sepsis pathophysiology despite not emerging as DEPs in the proteomic results [23]. Finally, sCD25 was studied as it has been described to be one of the eight criteria for HLH diagnosis according to the Histiocyte Society guidelines [7].

**Figure 6:**
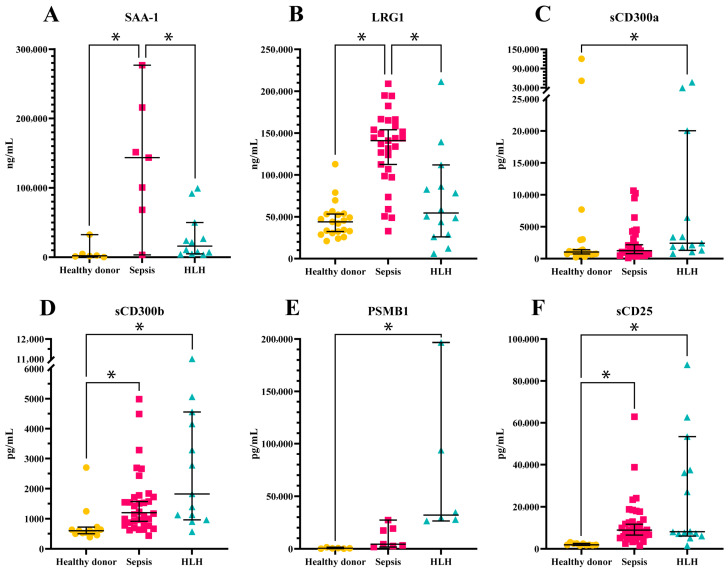
Validation results of candidate biomarkers in each group: Healthy donor (in yellow), Sepsis (in rose) and HLH (in blue). (**A**) SAA-1, (**B**) LRG1, (**C**) sCD300a, (**D**) sCD300b, (**E**) PSMB1, (**F**) sCD25. Bars show median and 95% confidence interval (CI) of the median. * *p* value < 0.05.

The validation results of the mentioned marker candidates are shown in Figure 6. (A) SAA-1 and (B) LRG1 were significantly upregulated in septic patients compared to both HLH patients and healthy donors. (C) sCD300a serum concentration was found to be significantly overexpressed in HLH compared to healthy donors. (D) sCD300b and (F) sCD25 showed a significant upregulation in both syndromes compared to healthy donors. Lastly, (E) PSMB1 was validated in a limited cohort of individuals and found to be significantly upregulated in HLH patients compared to healthy donors.

### 3.5. ROC Analysis of Candidate Biomarkers

**Figure 7:**
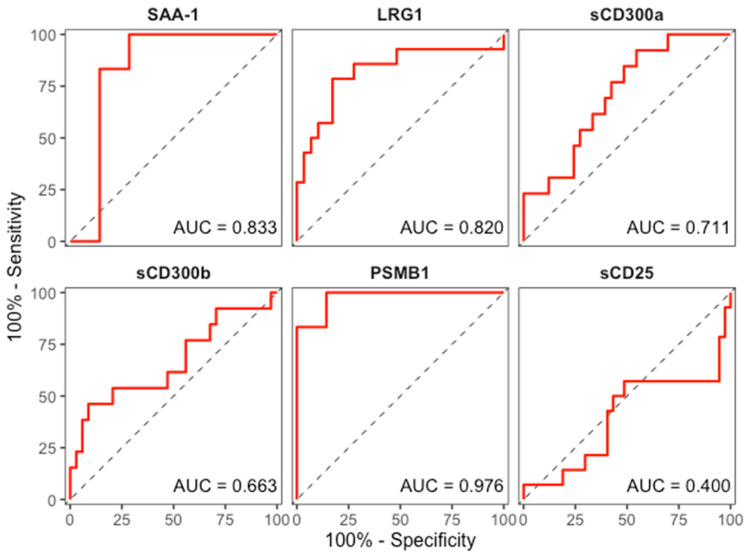
Receiver operating characteristic (ROC) curves for the validated biomarkers discriminating hemophagocytic lymphohistiocytosis (HLH) from sepsis. Curves are shown for SAA-1, LRG1, sCD300a, sCD300b, PSMB1, and sCD25 in the ELISA cohort.

To further assess the ability of the validated proteins to discriminate HLH patients from those diagnosed with sepsis, receiver operating characteristic (ROC) curves were generated for each marker using the ELISA cohort. The areas under the ROC curve (AUCs) were 0.711 for sCD300a, 0.663 for sCD300b, 0.820 for LRG1, 0.976 for PSMB1, 0.400 for sCD25 and 0.833 for SAA-1 (Figure 7). Thus, SAA-1 and LRG1 showed very good discrimination between HLH and sepsis, whereas PSMB1 displayed excellent discriminatory performance in this dataset. In contrast, sCD25 showed poor discriminatory capacity, consistent with its similar expression levels in HLH and sepsis, and sCD300a and sCD300b provided only modest separation between the two conditions.

## 4. Discussion

In our study, we validated a set of proteins that might facilitate the diagnosis of HLH and differential diagnosis between HLH and sepsis. A significantly different serum proteome signature was found between HLH and septic patients after the LC-MS/MS approach. It is worth mentioning that the analysis and interpretation of serum proteomes is sometimes difficult due to the presence of very-high-abundance serum proteins that can lead to the masking of possible candidate biomarkers, present in lesser concentrations [16]. For this reason, samples need to be depleted. However, in this study we show that analysis of the serum proteome from HLH, septic and healthy individuals is a novel and suitable strategy to ease the identification of new biomarkers for diagnosis of HLH and differential diagnosis between HLH and sepsis.

To our best knowledge, only two proteomic studies have been conducted to differentiate HLH from sepsis [6,24], of which only one [24] has been performed using a label-free proteomic approach, but the study was conducted with plasma samples. Moreover, to date, no biomarker is known as a gold standard for either diagnosis of each condition nor to differentiate both diseases [2,6].

According to our results, the serum concentration of SAA-1 and LRG1 could contribute to the differential diagnosis between HLH and sepsis. SAA-1, a major acute-phase reactant elevated in prolonged inflammatory conditions and previously described as a sepsis biomarker [25], was significantly upregulated in sepsis in comparison to HLH and healthy donors. Interestedly, the same pattern for SAA-1 was observed previously in plasma between sepsis and HLH patients [24]. A similar expression trend was found for LRG1, a protein released by activated neutrophils and hepatocytes, the function of which is not clearly known [25,26]. The downregulation of LRG1 in HLH likely reflected the reduced neutrophil counts found in some severe HLH patients [7]. Because both proteins are linked to the acute-phase response, their concentrations could, in principle, be influenced by demographic or clinical factors. However, in our cohort, serum samples were obtained at diagnosis before initiation of HLH disease- or sepsis-directed therapy, and Mann–Whitney U tests with Bonferroni correction did not reveal significant differences in SAA-1 or LRG1 levels between male and female patients or according to the presence of documented comorbidities. These findings suggest that, within the limits of our sample size, SAA-1 and LRG1 primarily reflect disease-related inflammation and might help discriminate between HLH and sepsis, although larger cohorts will be required to fully assess potential modulation by gender or comorbidities.

It is known that sCD25 is used as a marker of T-cell activation and it controls immunotolerance via T regulatory cells [27]. In our cohort, sCD25 was significantly upregulated in both HLH and sepsis compared with healthy donors, in line with previous reports [7,25]. These findings support its inclusion as one of the eight diagnostic criteria for HLH in the HLH-2004 clinical guidelines [7]. However, because sCD25 levels were similar in HLH and sepsis, our data indicate that sCD25 alone is not useful for differential diagnosis between these two hyperinflammatory syndromes. This underscores the need to interpret sCD25 in the context of the full clinical picture and additional laboratory parameters to improve discrimination between HLH and sepsis.

Apart from these acute-phase proteins, we analyzed sCD300a and sCD300b because they are receptors involved in the regulation of the inflammatory response through recognition of lipids and other ligands on immune cell surfaces [28,29]. We found that sCD300a was upregulated in HLH and not in septic patients. We hypothesize that sCD300a in HLH could be related to the fact that immune cells have the capacity to translocate this protein to the membrane in the presence of stimuli like LPS, IFN-γ and hypoxia [29]. These stimuli are known to be present in HLH [6] and, to our best knowledge, this is the first time that CD300a has been analyzed in HLH and sepsis. On the other hand, previous literature has shown that sCD300b is able to recognize ligands on the surface of macrophages to liberate inflammatory cytokines [23,29,30], and in two previous publications, a relationship between sepsis and sCD300b was described [23,29]. Our results revealed that sCD300b was upregulated in both HLH and septic patients. While our results confirm their involvement in hyperinflammatory states, the lack of differential regulation between HLH and sepsis suggests that sCD300a and sCD300b may not be suited for distinguishing the two conditions. However, CD300a could be useful for diagnosis of HLH.

Furthermore, PSMB1, a non-catalytic subunit of the 20S proteasome [31,32], was measured in this study. PSMB1 was found to be significantly upregulated in the serum of HLH patients when compared with healthy donors and non-significantly deregulated in HLH patients when compared with septic patients. Other studies confirmed that high levels of 20S proteasome are found in the serum of patients with different diseases [33], including sepsis, due to immunology activity [34]. These proteasomes have the same shape as intracellular ones and are totally active [35]. Although PSMB1 has a known role within homeostasis maintenance and inflammation, the mechanism of extracellular transport of this subunit and its role in this fluid are not known [36]. The elevation of PSMB1 in HLH patients may reflect an intensified demand for protein turnover, possibly driven by a higher activation of the immunoproteasome. The immunoproteasome, induced by inflammatory cytokines such as IFN-γ [37], modifies the proteolytic core of the proteasome to efficiently generate antigenic peptides for MHC class I presentation and is central to maintaining proteostasis under immune stress conditions [35]. Targeting this proteostasis machinery with drugs like Bortezomib, an FDA-approved proteasome inhibitor [22], has proven beneficial in other hyperinflammatory and malignant settings [38,39]. Hence, PSMB1 and related proteasome components may represent actionable therapeutic targets in HLH that need further research [40].

In line with the expression patterns, ROC curve analyses for the differential diagnosis between HLH and sepsis yielded excellent AUC values for PSMB1 (0.98) and very good values for SAA-1 and LRG1 (>0.8), indicating superb discriminatory performance in our cohort. By contrast, sCD300a and sCD300b achieved only modest AUCs, and sCD25 performed close to chance level, reinforcing that these markers are not suitable for distinguishing both diseases despite their elevation in hyperinflammatory states [7,25]. Taken together, these preliminary results suggest that SAA-1, LRG1 and particularly PSMB1 may form the core of a multi-marker panel to aid differential diagnosis. However, the development and validation of such combined models will require large cohorts and prospective evaluation before they can be translated into clinical practice.

Taking into account our results, we hypothesize that the disturbance in proteostasis, protein synthesis, and fatty acid oxidation observed in the deregulated pathway analysis for HLH could reflect an intense immune dysregulation that may affect multiple systems. The immunoproteasome, likely activated by elevated IFN-γ signaling and other inflammatory mediators [41], constantly degrades proteins to manage the antigenic load and sustain appropriate immunosurveillance. The persistent proteolysis, however, disrupts protein homeostasis, which triggers a substantial upregulation of transcriptional and translational machinery to resynthesize proteins and maintain cellular functionality. The observed enrichment in pathways related to translation initiation and SRP-dependent cotranslational targeting suggested that cells increase their protein synthesis capacity, striving to restore equilibrium against the ongoing protein turnover [42].

Such previously described processes are energetically demanding. To meet these requirements, cells may need more efficient energy sources, leading to augmented fatty acid β-oxidation. By degrading fatty acids, the organism gains the energy required to sustain the high immune, protein synthesis and degradation activities [43]. In essence, the severe inflammatory state in HLH appears to generate a coordinated response: immunoproteasome activation and heightened proteolysis trigger compensatory increases in protein production, which drive metabolic adaptations, including enhanced fatty acid oxidation, to sustain the energy-intensive effort of restoring intracellular homeostasis [44].

These insights not only deepen our understanding of HLH pathophysiology but also highlight new prospects for other therapeutic interventions in the future. Exploiting proteasome-targeting agents, such as Bortezomib and other next-generation inhibitors designed to modulate immunoproteasome activity, could offer innovative strategies to mitigate hyperinflammation and rebalance proteostasis, ultimately improving outcomes for patients with HLH.

## Figures and Tables

**Table 1:** Characteristics of the study cohort.

	Healthy Donors (n = 21)	Septic Patients (n = 37)	HLH Patients (n = 15)
**Gender**	10 females 11 males	21 females 16 males	11 females 4 males
**Median age (in years)**	6.54	3.66	2.92
**Comorbidities**			
Prematurity		2/37	
Neurological disease		4/37	2/15
Heart disease		3/37	
Chronic respiratory failure		3/37	1/15
HIV			1/15
Chronic renal failure			
**Organ dysfunction at diagnosis**			
Renal failure		7/37	3/15
Respiratory failure		10/37	7/15
Coagulopathy		6/37	6/15
Liver failure		3/37	11/15
Heart failure		34/37	1/15
Neurological failure		4/37	2/15
Thrombocytopenia		3/37	1/15

**Table 2:** Main overrepresented PPI clusters in HLH vs. healthy donors (HD) and HLH vs. sepsis.

Cluster	Biological Process GO Term	FDR	
		HLH vs. HD	HLH vs. Sepsis
1	Proteasomal ubiquitin-independent protein catabolic process (GO:0010499)	7.16 × 10^−16^	1.08 × 10^−14^
	Regulation of cellular amino acid metabolic process (GO:0006521)	6.04 × 10^−26^	1.08 × 10^−22^
2	SRP-dependent cotranslational protein targeting to membrane (GO:0006614)	1.08 × 10^−14^	8.79 × 10^−17^
	Translational initiation (GO:0006413)	1.38 × 10^−15^	1.46 × 10^−17^
3	Fatty acid beta oxidation using acyl-CoA oxidase (GO:0033540)	1.16 × 10^−7^	5.28 × 10^−5^
	Protein targeting to peroxisome (GO:0006625)	2.22 × 10^−9^	10^−6^

## Data Availability

The mass spectrometry proteomics data have been deposited into the ProteomeXchange Consortium via the PRIDE partner repository with the dataset identifier PXD063444 (https://www.ebi.ac.uk/pride) accessed on 29 April 2025.

## Supplementary Materials

The following supporting information can be downloaded at https://www.mdpi.com/article/10.3390/biomedicines13123113/s1: Table S1: Pathways predicted to be deregulated between HLH patients and healthy donors and between HLH patients and septic patients.

## Abbreviations

The following abbreviations are used in this manuscript:

HLH	Hemophagocytic lymphohistiocytosis
DEP	Differentially expressed protein
MCL	Markov cluster algorithm
GO	Gene ontology
IPA	Ingenuity pathway analysis
HD	Healthy donors
